# Mesenchymal Stem Cells Induce Expression of CD73 in Human Monocytes *In Vitro* and in a Swine Model of Myocardial Infarction *In Vivo*

**DOI:** 10.3389/fimmu.2017.01577

**Published:** 2017-11-20

**Authors:** Marta Monguió-Tortajada, Santiago Roura, Carolina Gálvez-Montón, Marcella Franquesa, Antoni Bayes-Genis, Francesc E. Borràs

**Affiliations:** ^1^REMAR-IVECAT Group, Health Science Research Institute Germans Trias i Pujol, Badalona, Spain; ^2^Department of Cell Biology, Physiology and Immunology, Universitat Autònoma de Barcelona (UAB), Barcelona, Spain; ^3^ICREC Research Program, Health Science Research Institute Germans Trias i Pujol, Badalona, Spain; ^4^Center of Regenerative Medicine in Barcelona, Barcelona, Spain; ^5^CIBERCV, Instituto de Salud Carlos III, Madrid, Spain; ^6^Nephrology Service, Germans Trias i Pujol University Hospital, Badalona, Spain; ^7^Cardiology Service, Germans Trias i Pujol University Hospital, Badalona, Spain; ^8^Department of Medicine, Universitat Autònoma de Barcelona (UAB), Barcelona, Spain

**Keywords:** CD73, adenosine, immunomodulation, mesenchymal stem cell, ectonucleotidase, myocardial infarction, purigernic signaling, regeneration

## Abstract

The ectoenzymes CD39 and CD73 regulate the purinergic signaling through the hydrolysis of adenosine triphosphate (ATP)/ADP to AMP and to adenosine (Ado), respectively. This shifts the pro-inflammatory milieu induced by extracellular ATP to the anti-inflammatory regulation by Ado. Mesenchymal stem cells (MSCs) have potent immunomodulatory capabilities, including monocyte modulation toward an anti-inflammatory phenotype aiding tissue repair. *In vitro*, we observed that human cardiac adipose tissue-derived MSCs (cATMSCs) and umbilical cord MSCs similarly polarize monocytes toward a regulatory M2 phenotype, which maintained the expression of CD39 and induced expression of CD73 in a cell contact dependent fashion, correlating with increased functional activity. In addition, the local treatment with porcine cATMSCs using an engineered bioactive graft promoted the *in vivo* CD73 expression on host monocytes in a swine model of myocardial infarction. Our results suggest the upregulation of ectonucleotidases on MSC-conditioned monocytes as an effective mechanism to amplify the long-lasting immunomodulatory and healing effects of MSCs delivery.

## Introduction

Exacerbated immune responses hamper solid organ transplantation and regeneration of injured tissues and lead to allergies, autoimmune diseases, and organ rejection. Antigen-presenting cells such as monocytes and dendritic cells (DCs) are responsible of linking the innate and adaptive phases of the immune response, providing signals to either trigger or downmodulate this response. Thus, in some clinical settings, modulation of monocyte polarization and/or DC maturation is one of the key points to prevent an unwanted immune response.

The immune response can be specifically modulated by signals from the millieu, including cytokines, chemokines, and others such as purinergic mediators. Among them, adenosine triphosphate (ATP) promotes inflammation when it is found in high amounts during apoptosis and necrosis in damaged tissues (1–3); on the contrary, removal of extracellular ATP avoids exacerbated tissue inflammation. Extracellularly, ATP is sequentially hydrolyzed to ADP and 5′AMP by the ectoenzyme nucleoside triphosphate diphosphohydrolase (NTPD-1/CD39) and to adenosine (Ado) by the ecto-5′-nucleotidase (ecto-5′-NT/CD73) (2, 4). The final product, Ado, is a powerful anti-inflammatory purine nucleoside, which has been described to immunosuppress macrophages, DCs, natural killer (NK), T, and B cells to promote tolerance (5, 6). Therefore, ATP hydrolysis is not only beneficial to reduce the pro-inflammatory ATP levels but also to produce the anti-inflammatory Ado. CD73 expression has been linked to the regulatory phenotypes of T and NK cells (6–8), and it is also a cell marker for progenitor or mesenchymal stem cells (MSCs) (9).

Mesenchymal stem cells have been widely associated with both regenerative and immunomodulatory capabilities (10–12). For instance, MSCs derived from umbilical cord MSCs (UCMSCs) (i.e., Wharton’s jelly) powerfully inhibit the inflammatory response of stimulated T cells (12). Also, cardiac adipose tissue-derived MSCs (cATMSCs), which represent a mesenchymal-like cell population with intrinsic cardiomyogenic potential (13), abrogate T cell proliferation upon stimulation with allogeneic mature monocyte-derived DCs (14) and promote tissue repair and immune suppression in an *in vivo* model of myocardial infarction (MI) (15, 16).

Among a broad number of mechanisms of action, MSCs generally modulate monocyte polarization toward an anti-inflammatory M2 phenotype (17–19) and restrain DC differentiation, resulting in the inhibition of the host immune response (20–22). At the same time, different *in vivo* models have demonstrated the need for monocytes/macrophages modulation to achieve healing and tissue repair by MSC treatment (19, 23, 24).

In this study, we first comparatively assess whether UCMSCs and cATMSCs (as distinct sources of MSCs) contribute to induce the functional expression of CD73 on monocytes, promoting the activation of their adenosinergic enzymatic activity. Finally, we evaluate the presence of infiltrated host monocytes expressing CD73 once cATMSCs are infused into swine post-infarcted myocardium.

## Materials and Methods

### Human cATMSC and UCMSC Isolation and Culture

The study protocols were approved by the Clinical Research Ethics Committee of our institution (Comitè Ètic d’Investigació Clínica, HuGTiP, Refs. CEIC: EO-10-13, EO-10-016 and EO-12-022), and conformed to the principles outlined in the Declaration of Helsinki. Written informed consent was obtained from donors.

Human cATMSCs were extracted from adipose tissue surrounding the base of the heart and around the aortic root from patients undergoing cardiothoracic surgery prior to coronary artery bypass graft initiation (*n* = 6), as reported in Bayes-Genis et al. (13) and Perea-Gil et al. (14). In addition, fresh umbilical cords (UC, *n* = 6) were obtained after birth and UCMSCs were isolated and cultured as reported in Monguió-Tortajada et al. (12).

### Monocyte Isolation

Peripheral blood mononuclear cells were obtained from leukocyte residues from healthy donors from the Blood and Tissue Bank (Barcelona, Spain) (*n* = 22) by Ficoll Hypaque Pus™ density gradient centrifugation (GE Healthcare Biosciences) at 1,800 rpm for 30 min, and CD3^+^ cells were depleted using the RosetteSep™ Human CD3 Depletion Cocktail (StemCell Technologies). Monocytes were then isolated using the EasySep™ Human anti-CD14 Positive Selection Kit (StemCell Technologies) or the MagniSort Human CD14 Positive Selection kit (eBioscience) following manufacturers’ instructions. Recovered cells were counted using PerfectCount Microspheres (Cytognos) and assessed for purity (>90% CD14^+^) and viability [≥93% by FSC/SSC and 7AAD^−^ (BD) gating] in a Canto II flow cytometer (BD).

### Monocyte Differentiation to DCs

Monocytes were cultured at 1 × 10^6^ cells/ml in complete medium composed of RMPI 1640 (Gibco) supplemented with 2 mM l-glutamine (Sigma), 100 U/ml penicillin (Cepa), 100 µg/ml streptomycin (Normon Laboratories), 5% human platelet lysate (Lonza), and DC differentiation cytokines: 300 IU/ml IL-4 and 450 IU/ml GM-CSF (Miltenyi Biotech). After 6 days, monocyte-derived dendritic cells (MDDCs) were harvested, counted, and assessed for viability and differentiation by 7AAD and CD11c staining, respectively (both from BD).

### Monocyte and MDDC Conditioning

Monocytes or MDDCs were cultured at 1 × 10^6^ cells/ml in a 20:1 ratio over a layer of cATMSCs or UCMSCs, which were previously let adhere to the culture plates for 4 h. As a control, monocytes were cultured alone or in the presence of 500 ng/ml LPS (Sigma). Contact dependency was assessed using a 24-well transwell system with 0.4 μm-pore polycarbonate membrane (Costar). Monocytes (4 × 10^5^) were seeded in each well and cATMSCs or UCMSCs (2 × 10^4^; 20:1 ratio) were applied to the upper chamber. Alternatively, monocytes were cultured in the presence of UCMSC conditioned media as published before (12).

After co-culture, monocytes or MDDCs were detached using accutase (Sigma) and washed with FACSFlow (BD) + 2% fetal bovine serum (FBS). Monocytes were stained with CD14-FITC and CD90-PE/Cy7 (BD) and separated by FACS in an Aria II sorter (BD). MDDCs were separated by FACS according to CD11c-Bv421^+^ (BD). Purity was always assured to be over 98%. Monocytes and MDDC were then pelleted and frozen at −80°C for whole RNA extraction.

Cell phenotype was assessed by incubation with CD11c-Bv421, CD14-FITC, CD25-PerCP/Cy5.5, CD39-Bv650, CD40-APC, CD73-PE, CD90-PE/Cy7, CD163-Bv711, CD206-PE-CF594 (BD), and/or CD80-PEVio770 (Miltenyi Biotech) or corresponding isotype control antibodies and acquisition in FACSCanto II and LSR Fortessa flow cytometers (BD). Analysis was performed using FlowJo X software.

### RNA Extraction and qPCR

Whole RNA content was isolated from cells using the RNeasy Mini kit (Qiagen). cDNA was synthesized using random hexamers (Qiagen) and the iScriptTM One-Step RT–PCR Kit (BioRad Laboratories) according to the supplier’s protocol. Analysis of the monocyte polarization markers expression was performed as described previously (12), using the primer sequences indicated in Table S1 in Supplementary Material. Alternatively, 8 µl of cDNA was preamplified with the TaqManW PreAmp Master Mix Kit (Applied Biosystems) in a final volume of 50 µl. Subsequently, 15 µl of preamplified cDNA were amplified in a final volume of 50 µl containing 25 µl TaqMan 2× Universal PCR Master Mix and 2 µl of the following FAM-labeled primer/probes (Applied Biosystems): CD73 (Hs00169777_m1), CD90 (Hs00264235_s1), and 18S (Hs99999901_s1). Data from four independent experiments were collected and analyzed on the LightCyclerW 480 Real-Time PCR System (Roche); each sample was analyzed in duplicate. The difference in threshold cycle 2(−ΔΔ*C_t_*) method was used to quantify the relative expression for each gene using 18S as endogenous reference (25).

### Cytokine Determination

The human IL10 and tumor necrosis factor α (TNFα) levels were measured in supernatants of 72 h-cultured monocytes using commercial ELISA kits (U-Cytech) following the manufacturer’s instructions.

### CD73 Enzyme Activity

Either MSCs or FACS-sorted conditioned monocytes were washed three times with MES buffer [0.025 M MES (Sigma) in 0.9% NaCl (Braun) pH 6.0] and cultured at 50,000 cells/well in the presence or absence of 5′AMP (Sigma) and the CD39 inhibitor POM1 (Tocris) or CD73 inhibitor APCP (ADP analog; Sigma) when indicated. After 2 h at 37°C, cells were centrifuged and supernatants harvested to freshly quantify Pi concentration using the malachite green phosphate colorimetric assay kit (BioVision), following the manufacturer’s instructions. 5′AMP and APCP alone were confirmed to yield negative values, and Pi production was calculated as the subtraction [Pi]_cells+5′AMP_ − [Pi]_cells alone_. Alternatively, the 2 h-supernatant was snap frozen at −80°C and analyzed for Ado concentration using the Adenosine assay kit (BioVision), following the manufacturer’s instructions.

### Animal Experimentation and Immunostaining

All animal studies were approved by the local Animal Experimentation Unit Ethical Committee (no. ES 100370001499) and complied with all guidelines concerning the use of animals in research and teaching as defined by the Guide for the Care and Use of Laboratory Animals (NIH Publication No. 80-23, revised 1996). Allogeneic porcine cATMSCs were isolated from cardiac adipose biopsy samples (average 0.4–5.9 g) from pigs undergoing cardiac surgery (*n* = 5), and processed as previously described (15). Succinctly, tissue specimens were washed in phosphate-buffered saline to remove contaminating debris and red blood cells, and digested in 0.015% collagenase (Type II-B, Sigma-Aldrich) for 30 min at 37°C in gentle agitation. The collagenase was inactivated by dilution with α-MEM containing 10% FBS, 2 mM glutamine, 1% penicillin–streptomycin (Gibco Invitrogen Corp.) (α-MEM-FCS). The cell suspension was centrifuged for 10 min at 1,200 × *g*, and the pellet was resuspended in α-MEM-FCS and filtered through a 100-µm mesh. Adhered cells were finally grown to subconfluence in α-MEM supplemented with 10% FBS, and cultured under standard conditions.

Engineered bioactive graft generation and implantation, MI induction, and immunohistological analysis was performed as described in previous studies (15, 16). In brief, a decellularized human pericardium-derived scaffold embedded with GFP-labeled porcine cATMSCs (treated animals; *n* = 7) or without cells (control animals; *n* = 7) was implanted covering the ischemic area in Landrace × Large White pigs 30 min after MI induction. In addition, a group submitted to the engineered bioactive graft enriched with cATMSCs but without MI induction was included (sham animals; *n* = 3). Animals were sacrificed after 30 days of follow-up.

Immunostaining was performed on cells grown in μ-dishes with glass bottom (Ibidi) or 10-µm myocardial sections against GFP, cardiac troponin I (Abcam), CD73, and CD163 (Novus Biologicals) Abs (1:100). Subsequently, secondary Abs (1:500) conjugated to Alexa Fluor 488, Alexa Fluor 594, Alexa Fluor 647 (Molecular Probes), Cy2 and Cy3 (Jackson ImmunoResearch Laboratories) were applied. Samples were finally counterstained with Atto 488-conjugated phalloidin (1:40) and/or 4′,6-diamidino-2-phenylindole dihydrochloride (1:10,000) (Sigma), and analyzed under an Axio Observer Z1 confocal microscope (Zeiss). For CD163^+^ and CD73^+^ cell quantification, at least four different optical fields from each section were measured under Image-Pro Plus software (6.2.1 version; Media Cybernetics, Inc.).

### Statistical Analysis

Values are expressed as mean + or ± SD. Kolmogorov–Smirnov analysis was used to check for normality of data and appropriate statistical tests are indicated for each dataset. Analyses were performed using the Graphpad Prism (6.0 version) and SPSS (21.0.0.0 version) softwares, and differences were considered significant when *p* < 0.05.

## Results

### Monocyte Polarization by cATMSCs and UCMSCs

**Figure 1:**
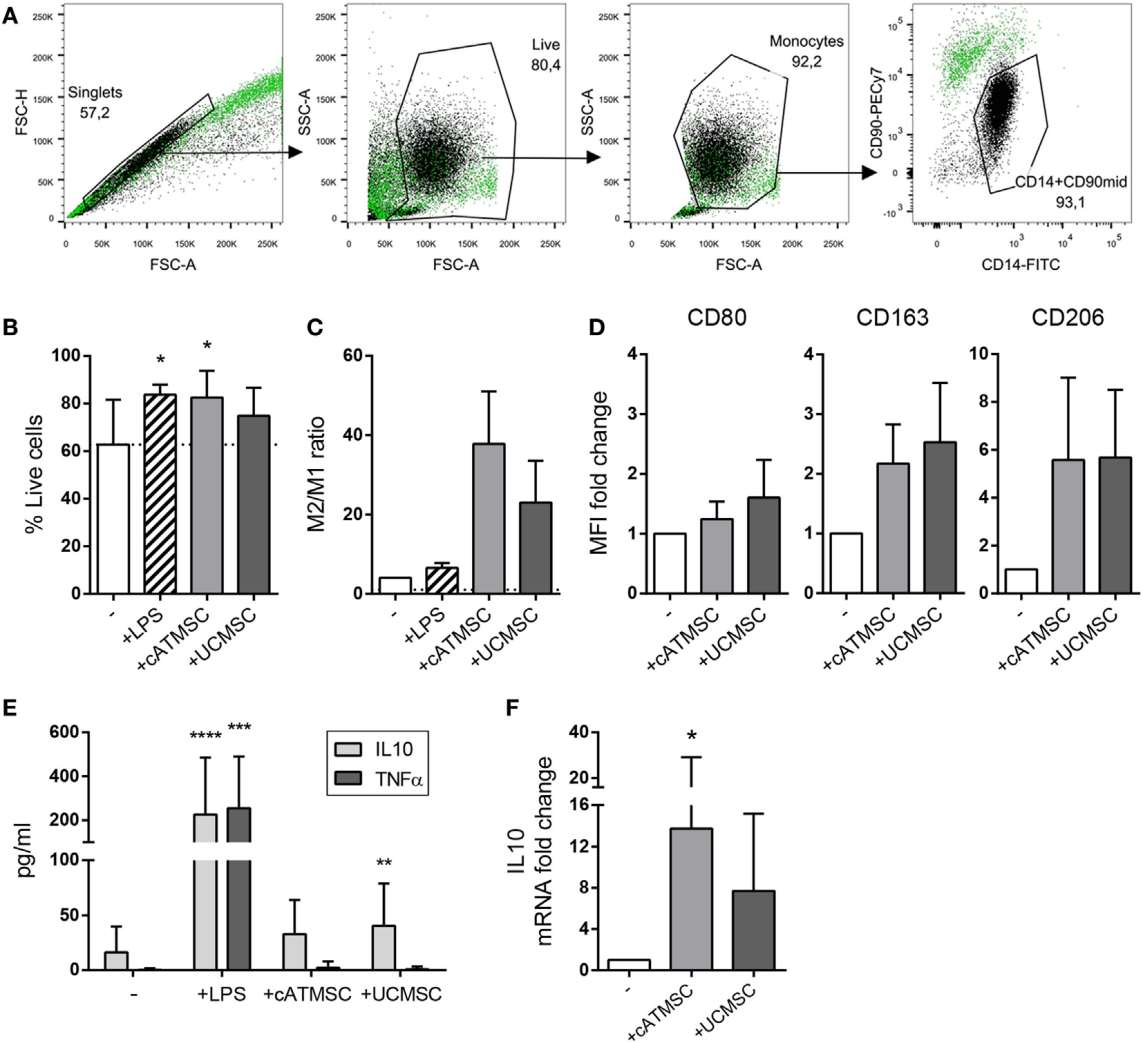
cATMSCs and UCMSCs skew monocytes toward an “M2” phenotype. **(A)** Monocytes (CD14^+^/CD90^mid^) were separated by FACS after co-culture with cATMSCs or UCMSCs (CD14^dim^/CD90^high^). A representative gating analysis of monocytes co-cultured with UCMSCs (black dots) compared to UCMSCs alone (green dots) is shown. **(B)** Viability of co-cultured monocytes. Data are mean + SD of 11 independent experiments. **(C)** FACS-sorted monocytes were checked for the expression of M1 and M2 markers by qPCR after 48 h of co-culture. Data are expressed as mean + SD of the ratio between the M2 (ΣmRNA fold change of CD163, CD206, TGM2, and CCL18) and M1 marker (mRNA fold change of CD80) depicted in Figure S1 in Supplementary Material. Data accounts for three independent experiments. **(D)** Fold increase in CD80, CD163, and CD206 MFI of monocytes cultured for 72 h with cATDPCs or UCMSCs, relative to monocytes alone (−). Data accounts for three independent experiments. **(E)** IL10 and TNFα cytokine levels in 72 h-culture supernatants. Data are mean + SD of 12 independent experiments. Statistical differences are indicated where *p < 0.05 by one-way ANOVA with Tukey’s *post hoc* test compared to monocytes cultured alone (−). **(F)** Fold increase in IL10 mRNA of monocytes cultured for 48 h with cATDPCs or UCMSCs, relative to monocytes alone (−). Data accounts for four independent experiments. cATMSCs, cardiac adipose tissue-derived MSCs; UCMSCs, umbilical cord MSCs; TNFα, tumor necrosis factor α.

To investigate the effect of MSC conditioning, we first analyzed the capacity of different human MSCs to skew monocyte polarization *in vitro*. Monocytes were co-cultured for 48 h with either cATMSCs or UCMSCs, and LPS as a positive control of monocyte activation. After co-culture, monocytes (CD14^+^/CD90^mid^) were easily isolated from MSCs (CD14^dim^/CD90^high^) by FACS according to both their CD14 expression and lower expression of CD90 compared to MSCs (Figure 1A). Monocytes exhibited better viability when co-cultured with MSCs (Figure 1B).

Both cATMSC and UCMSCs managed to upregulate the M2 markers CD163, CD206, TGM2, and CCL18 in monocytes at the mRNA level (Figure S1 in Supplementary Material), while the M1 marker CD80 remained unchanged. On the contrary, LPS activation of monocytes led to the increased expression of CD80. The relative mRNA expression of all markers studied (Figure 1C), suggested that both MSCs were promoting an M2 phenotype in monocytes. This phenotype was further assessed by surface protein expression, and while CD80 was unchanged, CD163 and CD206 did increase when monocytes were co-cultured with MSCs (Figure 1D). Furthermore, the cytokine profile showed that MSCs promoted the secretion of IL10 by monocytes, while no TNFα was detected (Figure 1E). The increased IL10 mRNA transcription of sorted monocytes after co-culture with MSCs (Figure 1F) together with the undetectable IL10 in MSC’s supernatants (data not shown) could attribute IL10 production to monocytes, further confirming an anti-inflammatory profile induced by MSC co-culture.

### Induction of CD73 Expression in Monocytes

After confirming the M2 skewing capabilities of cATMSCs and UCMSCs toward monocytes, we next studied the expression of adenosinergic ectoenzymes on these cells.

**Figure 2:**
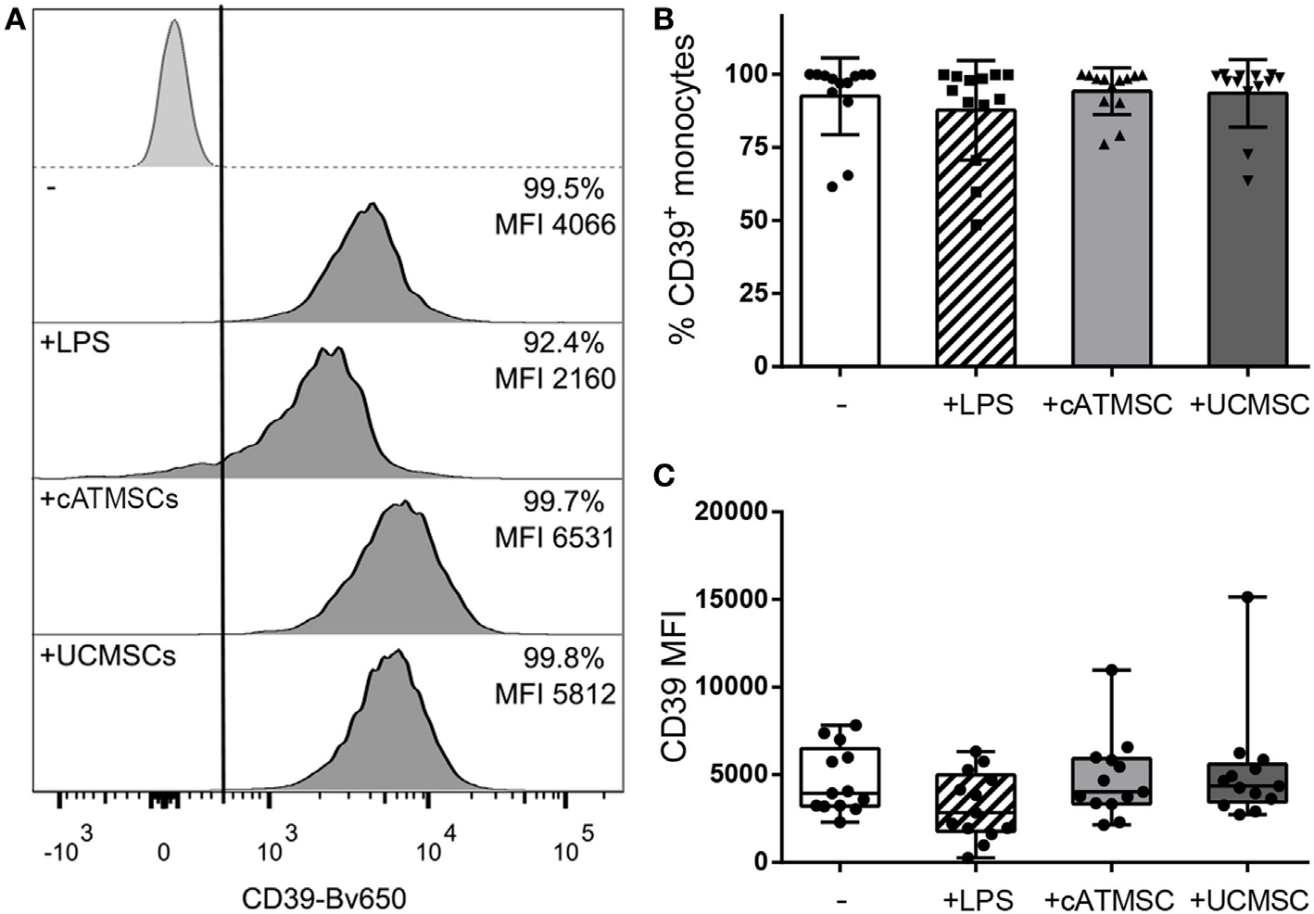
CD39 expression is maintained in monocytes co-cultured with MSCs. **(A)** Representative histograms depicting the CD39 expression of monocytes cultured for 72 h alone (−) or with LPS, cATMSCs, or UCMSCs. The isotype control is depicted in the top row; the % of positive cells and the MFI for the total monocyte population (CD14^+^/CD90^mid^) are indicated in each plot. **(B)** Percentage of CD39^+^ and **(C)** CD39 MFI of monocytes cultured for 72 h alone (−) or with LPS, cATMSCs or UCMSCs. Data account for 12 independent experiments. cATMSCs, cardiac adipose tissue-derived MSCs; UCMSCs, umbilical cord MSCs.

CD39 was already present in fresh blood peripheral blood monocytes (data not shown) and remained highly expressed on monocytes cultured alone or in the presence of LPS, cATMSCs, or UCMSCs (Figure 2).

**Figure 3:**
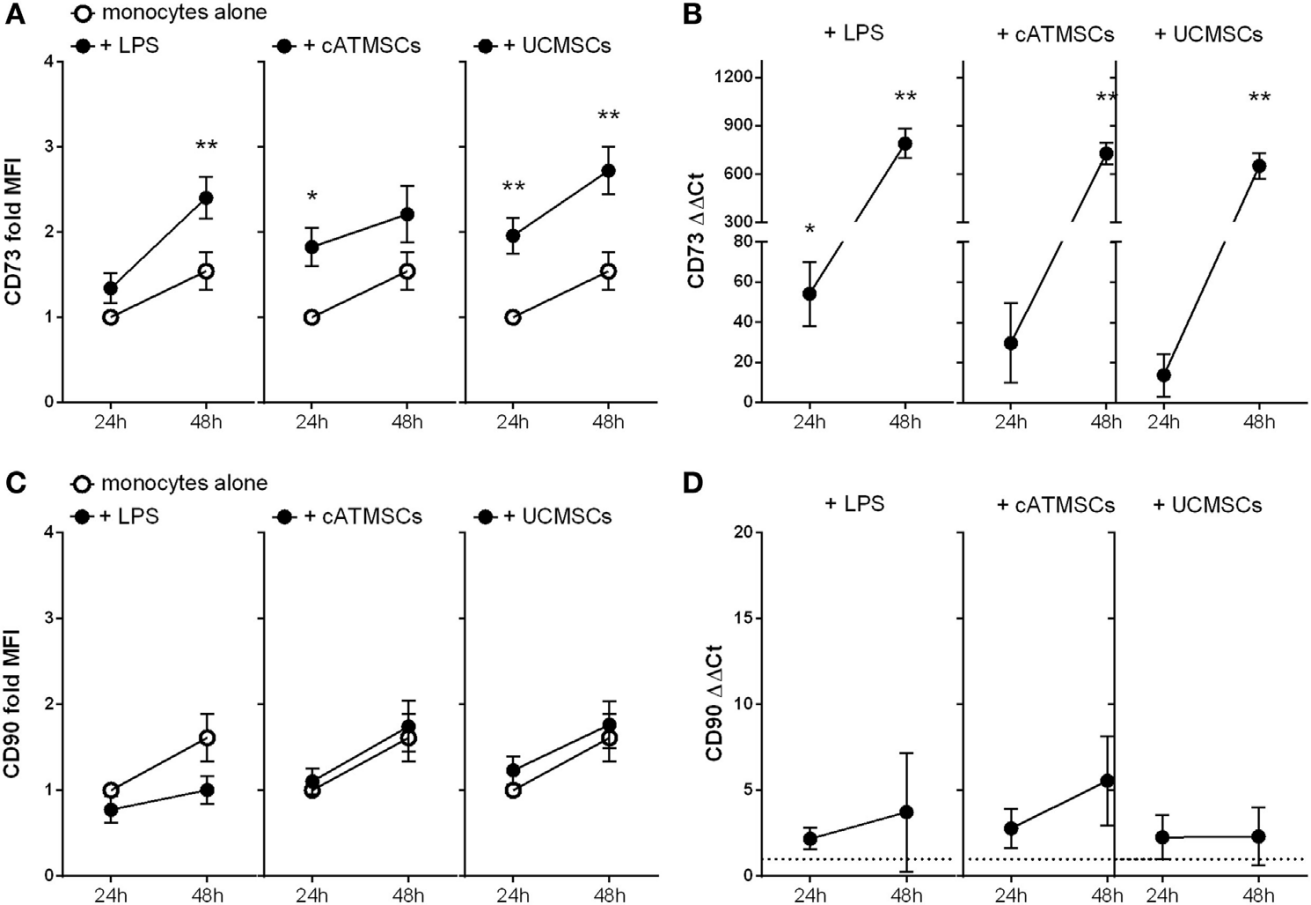
CD73 is induced while CD90 remains unchanged in monocytes co-cultured with cATMSCs and UCMSCs. **(A,C)** Fold increase in CD73 and CD90 MFI of monocytes cultured for 24 or 48 h with LPS, ATDPCs, or UCMSCs (black dots), relative to 24 h-cultured monocytes alone (white dots). Statistical differences are indicated where *p < 0.05 and **p < 0.01 by two-way ANOVA compared to monocytes cultured alone. **(B,D)** Fold increase in CD73 and CD90 mRNA (ΔΔ*C_t_*) of monocytes, relative to monocytes cultured alone. Statistical differences are indicated where *p < 0.05; **p < 0.01; and ***p < 0.001 by one-sample *T* test. Data are expressed as mean ± SD and accounts for four independent experiments of different monocyte and MSC donors. MSC, mesenchymal stem cell; cATMSCs, cardiac adipose tissue-derived MSCs; UCMSCs, umbilical cord MSCs.

Interestingly, monocytes cultured on a layer of both types of MSCs expressed higher levels of CD73 protein at 24 and 48 h of culture compared to control (Figure 3A). CD73 mRNA was incremented more than 500 times in monocytes after 48 h of co-culture with either cATMSCs or UCMSCs in comparison to cultured alone (Figure 3B), pointing to the induction of protein expression. Of note, LPS activation of monocytes also incremented CD73 expression. As a negative control, another classical MSC marker, CD90, was analyzed in conditioned monocytes. CD90 was found to be unchanged both at a protein and RNA levels (Figures 3C,D), suggesting the apparent specificity to CD73 acquisition and absence of a trogocytosis-like phenomenon.

**Figure 4:**
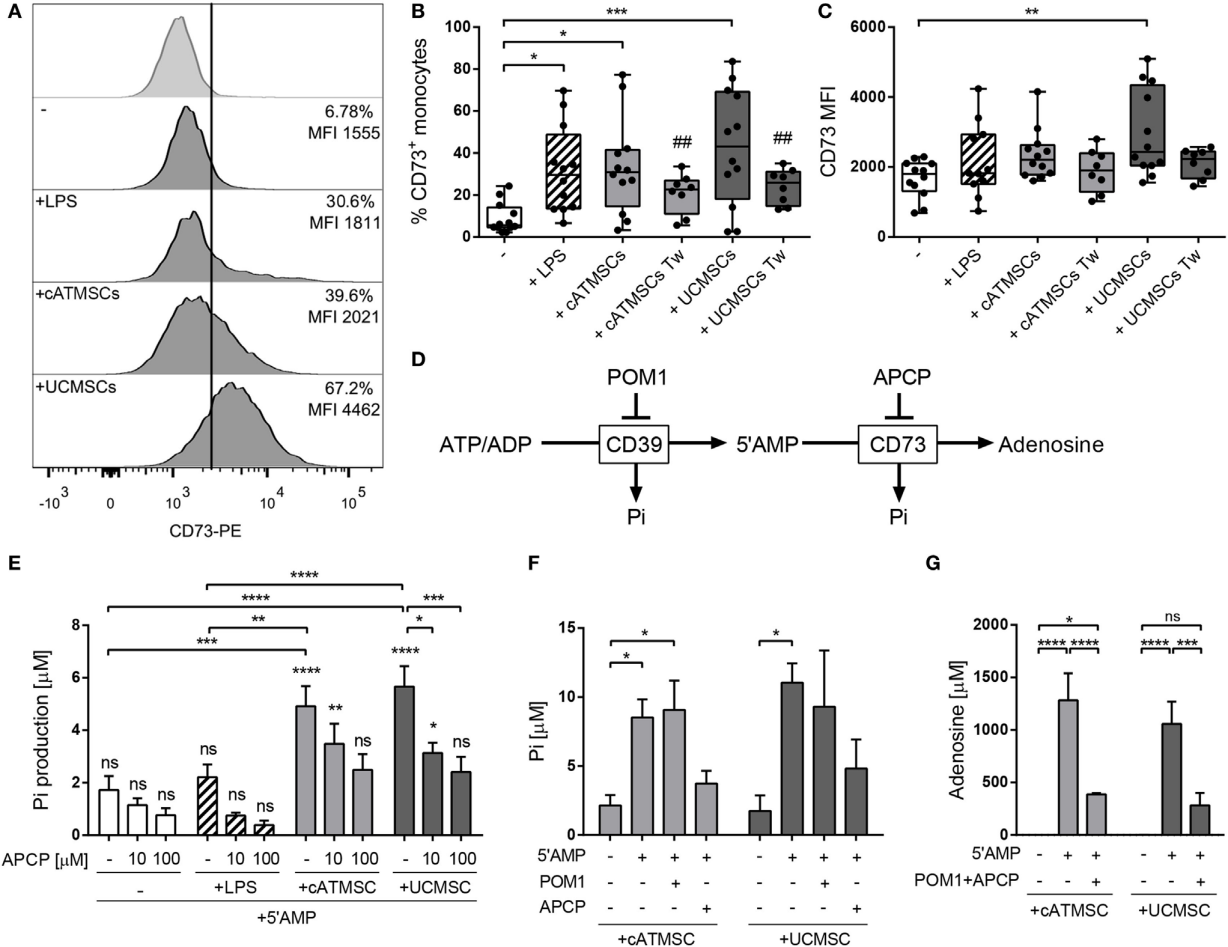
Monocytes co-cultured with cATMSCs or UCMSCs upregulate the adenosinergic enzymatic activity. Monocytes cultured for 72 h alone (−) or with LPS, cATMSCs or UCMSCs were checked for CD73 surface expression and activity. **(A)** Representative histograms of CD73 expression. The isotype control is depicted in the top row; the % of positive cells and the MFI for the total monocyte population (CD14^+^/CD90^mid^) are indicated in each plot. **(B)** Percentage of CD73^+^ and **(C)** CD73 MFI of monocytes as mean ± SD of 12 independent experiments. Tw: co-culture in transwell system. Statistical differences are indicated where *p < 0.05, **p < 0.01, and ***p < 0.001 by one-way ANOVA with Tukey’s *post hoc* analysis. **(D)** Schematic representation of the enzymatic hydrolysis of ATP/ADP to 5′AMP by CD39 and to Ado by CD73, which can be inhibited by POM1 and APCP, respectively. Inorganic phosphate (Pi) is produced as a byproduct in each step. **(E)** Levels of inorganic phosphate (Pi) produced by FACS-sorted monocytes analyzed after the addition of the CD73 substrate (5′AMP; 1 mM) with or without the CD73 inhibitor (APCP). Data are presented in bars as the mean + SD concentration of Pi subtracted from the Pi present in monocytes without 5′AMP. Data account for 9–13 independent experiments. **(F,G)** Levels of inorganic phosphate (Pi) **(F)** and of Ado **(G)** produced by FACS-sorted monocytes analyzed after the addition of the CD73 substrate (5′AMP; 1 mM) with or without the CD39 inhibitor (POM1) or the CD73 inhibitor (APCP). Data are presented in bars as the mean + SD concentration of Pi. Data accounts for four independent experiments. Statistical differences in each bar are compared to cells without 5′AMP or to the indicated groups where *p < 0.05, **p < 0.01, ***p < 0.001, and ****p < 0.0001 by two-way ANOVA with Tukey’s *post hoc* analysis. cATMSCs, cardiac adipose tissue-derived MSCs; UCMSCs, umbilical cord MSCs; ATP, adenosine triphosphate; Ado, adenosine.

As high levels of CD73 mRNA were detected after 48 h of conditioning, the CD73 protein expression and functional activity was further evaluated at 72 h of co-culture. Monocytes co-cultured with both cATMSCs and UCMSCs expressed higher levels of CD73 on the surface in comparison to control monocytes and LPS-stimulated monocytes (Figures 4A–C).

We also investigated whether direct cell contact between MSCs and monocytes was necessary to induce CD73 expression. For that purpose, co-culture experiments were performed using transwell culture plates, in which monocytes did not increase CD73 expression to the same extent compared to direct co-culture with MSCs (Figures 4B,C). To corroborate such observations, monocytes were cultured in the presence of MSC’s conditioned medium (Figure S2 in Supplementary Material), which neither induced CD73 expression in monocytes.

### CD73 Induced in Conditioned Monocytes Is Functional

The functionality of CD73 was then evaluated through the production of inorganic phosphate (Pi) and Ado yielded from the hydrolysis of the CD73 substrate 5′AMP (Figure 4D). Confirming the high upregulation of CD73, MSC-co-cultured monocytes induced a much greater amount of Pi production compared to control and also LPS-stimulated monocytes (Figure 4E). Pi production was only detected when 5′AMP was added to the cells, as an indication of CD73 action rather than from other ectoenzymes. Enzyme activity inhibition was also evaluated by using the ADP analog APCP. These experiments showed a dose-dependent abrogation of Pi production by MSC-conditioned monocytes and a fully blocked Pi production in LPS-stimulated monocytes (Figure 4E).

In parallel experiments, CD73 enzyme functionality was also confirmed in cATMSCs and UCMSCs as a positive control. As expected by their higher CD73 expression, both cATMSCs and UCMSCs displayed a higher 5′AMP hydrolytic activity which was dose-dependently blocked with APCP (Figure S3 in Supplementary Material).

To further discard the production of Pi from other upstream hydrolysis, we checked the Pi concentration in the presence of the CD39 inhibitor POM1 (Figure 4F). Again, Pi was only produced in the presence of the CD73 substrate 5′AMP, and while POM1 did not affect Pi production by MSC co-cultured monocytes, it was inhibited when APCP was added (Figure 4F).

We then confirmed the production of Ado by MSC co-cultured monocytes, which was found in the supernatant only when 5′AMP was added to the monocytes and was abrogated by APCP addition (Figure 4G), indicating a CD73 functional enzymatic action.

### MDDCs Are Not Affected by MSC Conditioning

**Figure 5:**
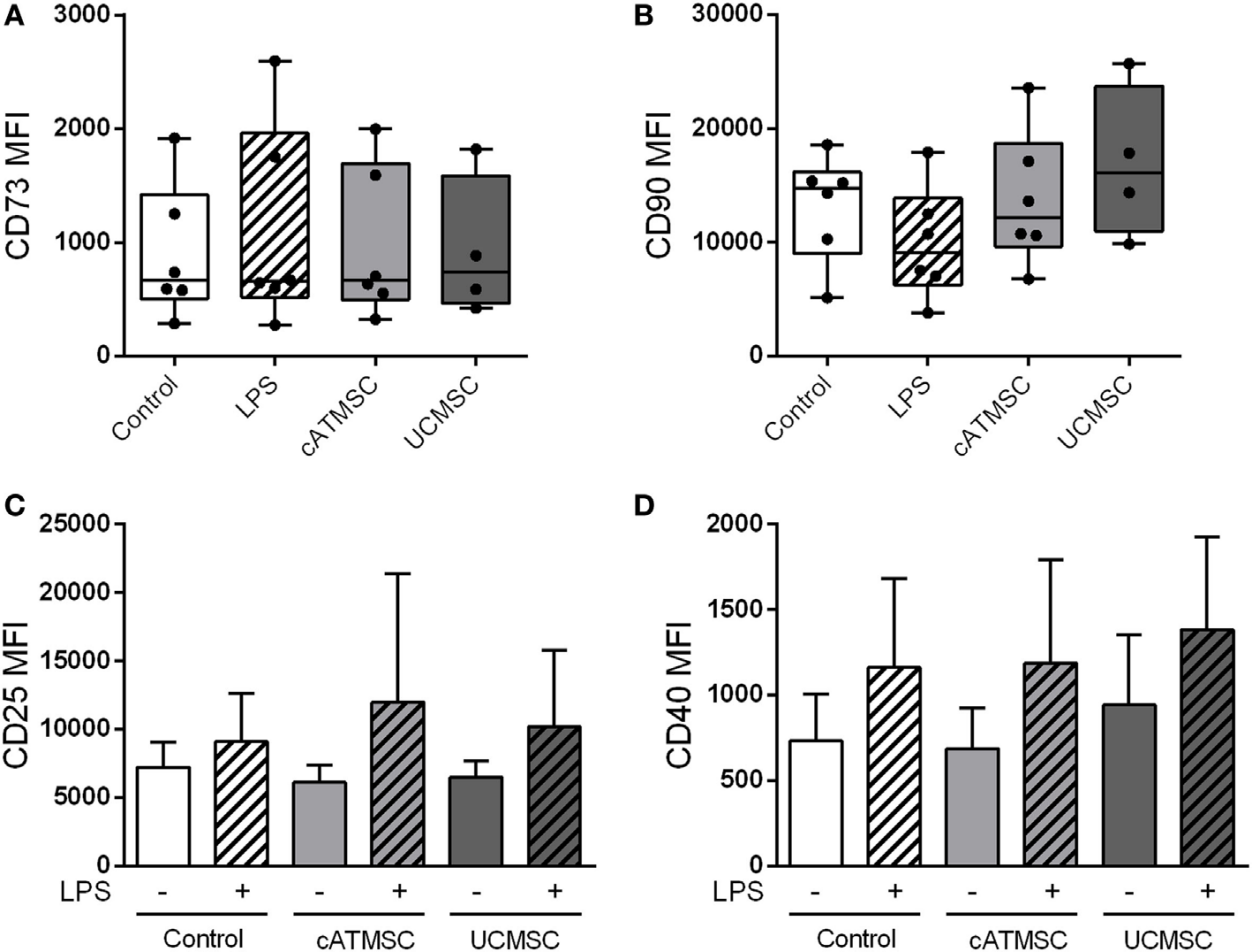
CD73 and CD90 are not induced by MSC co-culture in MDDCs. **(A,B)** CD73 and CD90 MFI of MDDCs cultured for 48 h alone or with LPS, ATDPCs, or UCMSCs. **(C,D)** MFI values of the maturation markers CD25 and CD40 of MDDCs cultured for 48 h with or without cATMSCs or UCMSCs, and further stimulated with LPS (dashed bars) or not. Data are expressed as mean + SD and accounts for six independent experiments. MSC, mesenchymal stem cell; cATMSCs, cardiac adipose tissue-derived MSCs; UCMSCs, umbilical cord MSCs; MDDCs, monocyte-derived dendritic cells.

In light of monocyte modulation by MSCs, we sought to explore the ability of MSCs to alter the maturation and CD73 expression of further differentiated cells such as MDDCs. Opposed to monocytes, the levels of CD73 and CD90 remained unchanged in MDDCs co-cultured with cATMSCs or UCMSCs (Figures 5A,B), which was confirmed as well by studying the mRNA levels of both CD markers (data not shown). On the other hand, MSCs failed to modulate MDDC maturation markers CD25 and CD40 (Figures 5C,D), which were upregulated to the same extent by LPS stimulation, regardless of MSC co-culture.

### Host Monocytes Acquire CD73 *In Vivo* in Swine Post-Infarcted Myocardium Treated with Porcine cATMSCs

To investigate whether this *in vitro* effect occurred *in vivo*, we analyzed the presence of infiltrating monocytes and CD73 expression in a swine model of MI locally treated with porcine cATMSCs, previously reported by our group to reduce the amount of infiltrating effector T cells in the infarcted tissue and to ameliorate the regeneration of the myocardium (15, 16). In these studies, treated animals vs. controls experimented a significant reduction in infarct size (3.4 ± 0.6 vs. 6.5 ± 1%; *p* = 0.015) and fibrosis in the infarct scar (collagen I/III ratio; 0.49 ± 0.06 vs. 1.66 ± 0.5; *p* = 0.019), and improved in cardiac function (left ventricular ejection fraction; 7.5 ± 4.9 vs. 1.4 ± 3.7%; *p* = 0.038, and stroke volume; 11.5 ± 5.9 vs. 3 ± 4.5 ml; *p* = 0.019).

**Figure 6:**
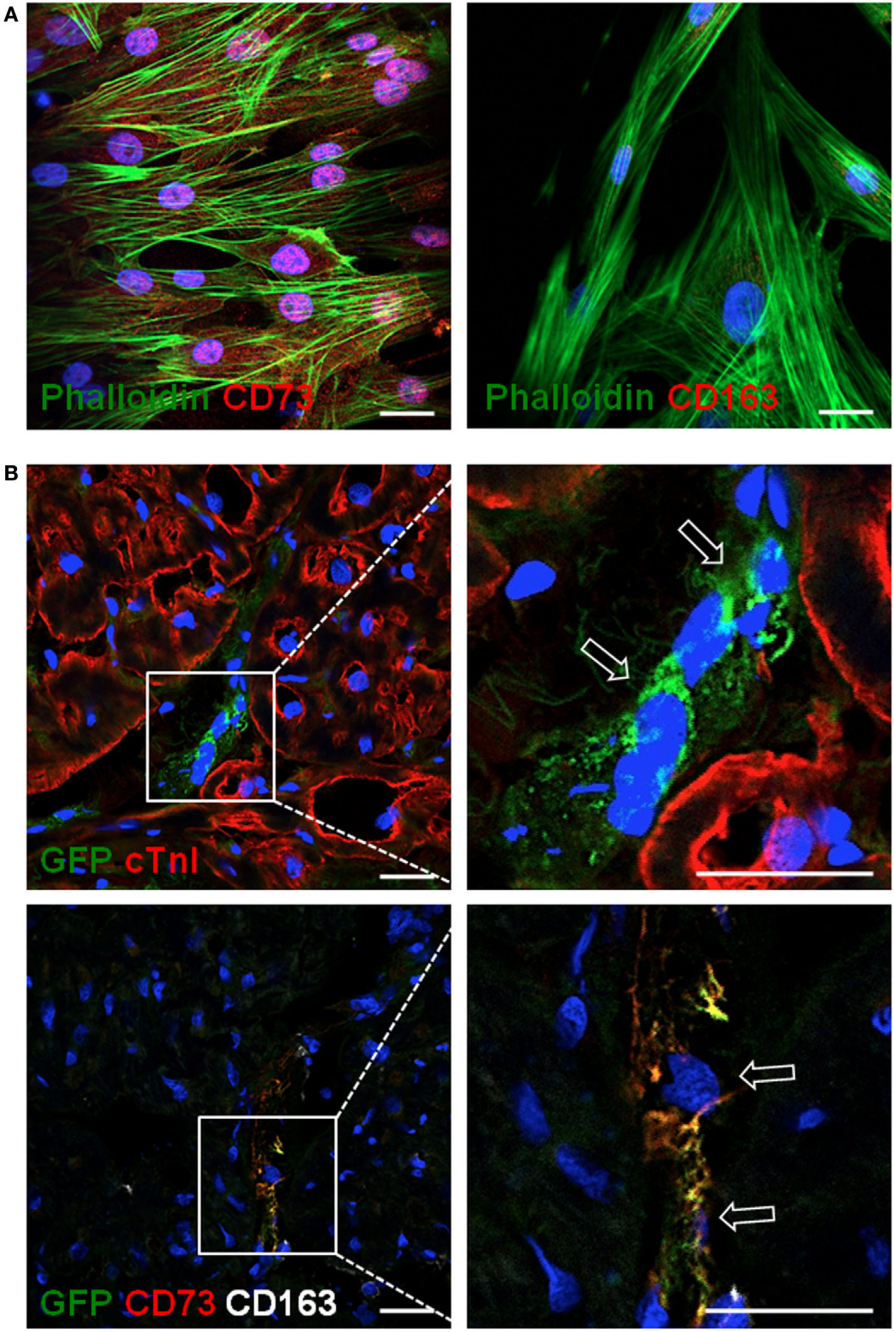
Allogeneic cATMSCs migrated to the infarcted myocardium after graft implantation in swine. Representative confocal microscope images showing **(A)** porcine cATMSCs in *in vitro* culture or **(B)** sections within the infarcted myocardium. **(A)** Porcine cATMSCs are positive for CD73 (left, red) and negative for CD163 (right, red). **(B)** Presence of GFP^+^ porcine cATMSCs (empty arrows) in post-infarcted myocardium, also positive for CD73 but negative for CD163 (lower panels). Cell morphology, cardiac muscle, and cell nuclei are also counterstained using Atto 488-phalloidin, anti-cTnI Ab, and DAPI, respectively. Scale bars = 50 µm. cATMSCs, cardiac adipose tissue-derived MSCs; DAPI, 4′,6-diamidino-2-phenylindole dihydrochloride.

**Figure 7:**
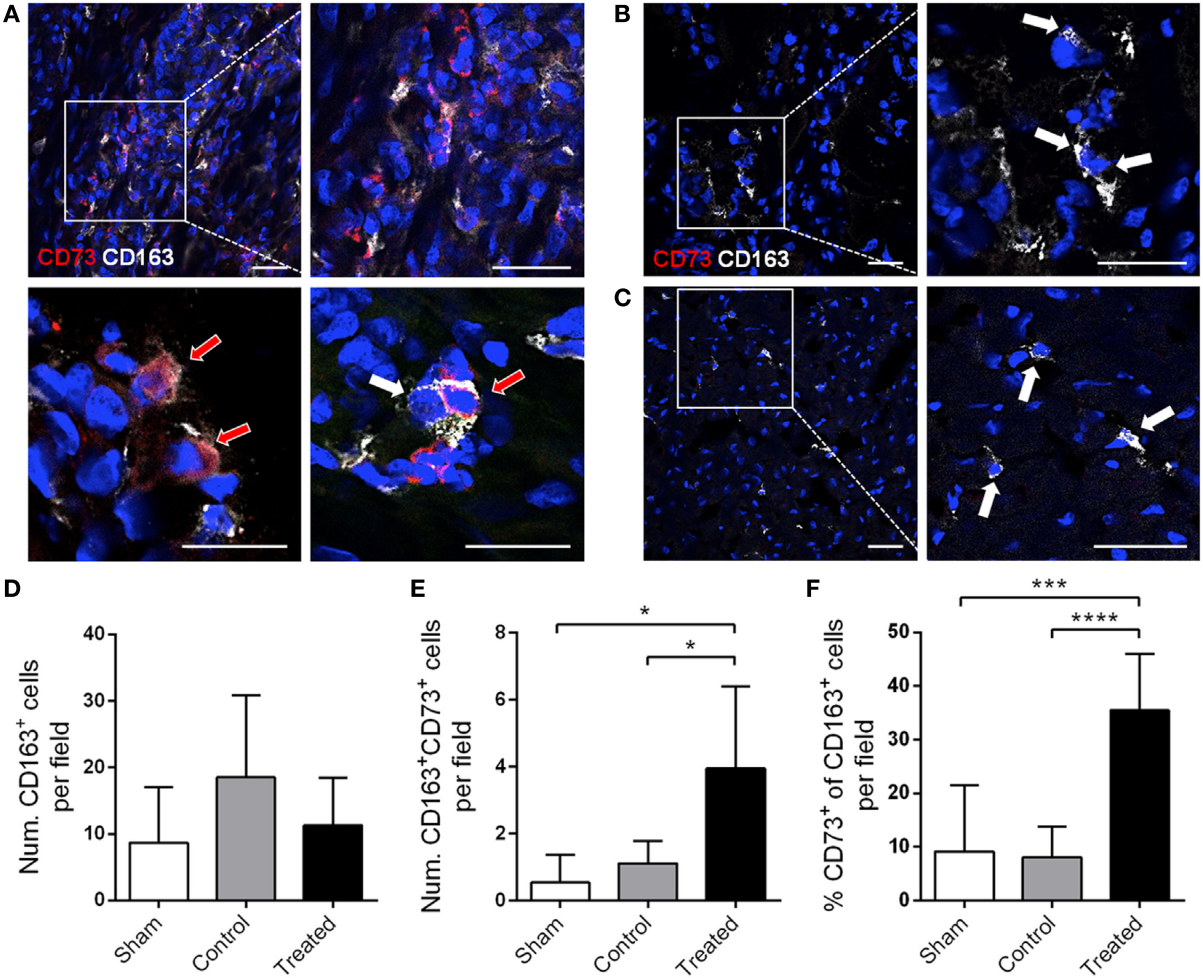
CD73 is also induced *in vivo* in host monocytes in swine post-infarcted myocardium treated with porcine cATMSCs. **(A–C)** Infiltrating CD163^+^ monocytes (white arrows), some of them expressing CD73 (red arrows) in a cATMSC-treated animal **(A)**, and CD163^+^ monocytes (white arrows) in a control **(B)** and sham **(C)** animals, both negative for CD73. CD73 is shown in red and CD163 in white, and nuclei are counterstained with DAPI in blue. Scale bars = 50 µm. **(D,E)** Histograms showing the number of CD163^+^ monocytes **(D)**, CD73^+^ CD163^+^ monocytes **(E)** per optical field in all studied groups. **(F)** Percentage of CD73^+^ monocytes out of the CD163^+^ monocytes. Differences in immunohistochemical quantifications were compared using one-way ANOVA for multiple comparisons, with Tukey’s test for the *post hoc* analysis. cATMSCs, cardiac adipose tissue-derived MSCs; DAPI, 4′,6-diamidino-2-phenylindole dihydrochloride.

Mesenchymal stem cells were delivered in the ischemic area by the implantation of an allogeneic engineered bioactive graft comprising GFP-labeled porcine cATMSCs, compared to the use of an “empty” graft in control animals. In this study, 1 month after the implantation, we detected how GFP^+^ cells (cATMSCs), expressing CD73 but not the monocyte marker CD163 (Figures 6A,B), actively migrated from the graft to the infarcted tissue and persisted within the damaged area in treated animals. Noticeably, treated, control, and sham animals had infiltrating monocytes (CD163^+^) in the infarcted tissue (Figures 7A–C), but only in treated animals receiving cATMSCs after MI the infiltrating host monocytes gained CD73 expression. After quantification, no differences were found in the amount of CD163^+^ infiltrating monocytes between groups (Figure 7D). However, we could corroborate the gain on CD73 expression on host monocytes in treated vs. both control and sham animals in terms of absolute numbers (3.95 ± 2.45 vs. 1.12 ± 0.66 vs. 0.54 ± 0.83 CD73^+^ CD163^+^ cells; *p* = 0.028 and *p* = 0.019, respectively) (Figure 7E), and also in the percentage of CD73^+^ out of CD163^+^ monocytes (35 ± 11 vs. 8 ± 6 vs. 9 ± 12%; *p* = 0.001 and *p* = 0.0001, respectively) (Figure 7F).

## Discussion

In this work, we demonstrate the molecular interplay between MSCs and monocytes and highlight the induction of the adenosinergic pathway on monocytes as an additional mechanism of immunomodulation and tissue healing. Particularly, we show that both human UCMSCs and human cATMSCs induce specifically in monocytes expression of the CD73 ectonucleotidase. By this function, together with the constitutive expression of CD39, monocytes would gain the ability to sequentially hydrolyze ATP to increase extracellular Ado concentration. Moreover, we corroborate this effect *in vivo*, as the local delivery of porcine cATMSCs into post-infarcted swine myocardium effectively resulted into the expression of CD73 on infiltrating host monocytes, fostering an anti-inflammatory milieu.

The degradation of exogenous pro-inflammatory ATP into anti-inflammatory Ado has been attributed a key role for the control of inflammation in the local environment, and also to have systemic effects (5, 6, 26, 27). Ado reduces NK, macrophage, dendritic, B and T cells activation (4, 28–30), neutrophil accumulation (31), while promotes the generation of M2 macrophages and expansion of regulatory T cells (CD39^+^/CD73^+^), thus establishing an adenosinergic amplification loop (5, 32–35). It further proves to be a powerful systemic immunosuppressant as Ado production is used as an immune evasion strategy by pathogens (36–38) and cancer cells (8, 39, 40), and its accumulation leads to a severe combined immunodeficiency in patients lacking ADA for Ado degradation (41, 42). Remarkably, Ado production also helps in wound healing and tissue repair (43), as inhibits post-hypoxic vascular leakage (44) and promotes angiogenesis *via* VEGF production by macrophages (45). These effects have been proven *in vivo*, where increased Ado levels reduced necrotic injury and edema formation, and limited infarct size in a mouse and swine models of MI (46, 47).

The presence of the purinergic ectoenzymes CD39/CD73 has been described for different organs and cell types. For instance, while MSCs and endothelial cells display a constitutive expression (48, 49), CD39/CD73 are triggered upon cell activation in the generation of Tregs, whereas CD73 is reduced in activated B cells (50). MSCs have also been shown to induce CD73 expression in NK cells, although to a much lesser extent than we observed in monocytes (7). In our experiments, we interestingly observed that LPS stimulation of TLR4 incremented CD73 expression in monocytes, probably as a balancing mechanism of cell activation, as was observed in mouse before (45, 51). However, only MSC co-culture lead to sustained CD73 expression and to active 5′AMP hydrolysis by these cells, in a mechanism dependent of cell contact. Monocytes were polarized by both cATMSCs and UCMSCs toward an anti-inflammatory M2-like phenotype and to secrete immunomodulatory cytokines (IL10, CCL18) (17, 52, 53), but it is worth to mention that other M2 switches such as the classical “M2A” induction by IL4 does not promote CD73 upregulation (54).

These observations are important given the reported short lifespan of MSCs after infusion *in vivo*. Once injected i.v., MSCs get trapped in the lung barrier because of their big size and are removed by monocytes/macrophages within hours (55–58), thus theoretically impeding the action of MSCs in the target tissue. Nevertheless, MSCs still promote a long-lasting systemic immunosuppressive effect for the resolution of inflammation and regeneration of wounded tissue. These effects could be firstly mediated temporarily by paracrine mediators, but secondarily by the modulation of the host’s immune cells. In this sense, soluble molecules such as interleukin 6, prostaglandin E2, transforming growth factor-β, indoleamine 2,3-dioxygenase, hepatic growth factor, human leukocyte antigen-G, TSG6, and extracellular vesicles have been attributed to promote such paracrine effects (12, 58, 59). Moreover, given that MSCs express CD39 and CD73 constitutively, Ado production is also part of MSC’s paracrine immunosuppressive activity. In fact, CD73 itself can also have systemic effects as it can be both shed from the membrane and act in its soluble form (60–62) and also be released within extracellular vesicles, as proved by Amarnath et al. in an animal model of Th1 inflammation treated with infused MSCs, in which systemic Ado production by released CD73 mediated the resolution of inflammation (63).

Following these MSC paracrine primary immunomodulatory effects, MSCs may modulate the action of immune cells for the generation of regulatory environments. Monocytes, which are present in great numbers especially in the lungs, would interact with the infused MSCs and acquire an M2-like phenotype, including CD73 mRNA expression in less than 24 h, as shown by our results. These CD39^+^/CD73^+^ monocytes are fit to easily go through the lung barrier and migrate to the inflamed tissue to promote *in situ* immunomolulation and healing. Although the level of CD73 activity was much higher in MSCs, CD39^+^/CD73^+^ monocytes would have the advantage of migration and delivering a targeted local effect.

At the same time, when MSCs are delivered directly to the injured tissue, they can regulate monocyte function locally, which are, together with granulocytes, the first cells to infiltrate into inflamed tissue (64). Remarkably, we previously demonstrated the use of a scaffold of decellularized human pericardium for the local delivery of porcine cATMSCs to the post-MI injured tissue. MSC treatment attained *in vivo* attenuation of inflammation (i.e., fewer activated T cells) and promoted the regeneration of the damaged myocardial tissue in post-infarcted pigs (16). Here, we also show the acquisition of CD73 expression by infiltrated host monocytes in MSC-treated animals, thus establishing an adenosinergic positive loop. The gain in CD73 by host monocytes was dependent on the combination of monocyte activation in response to pro-inflammatory damage-associated molecular patterns released after MI and the modulation by cATMSCs contained within the graft, given that both control and sham animals lacked presence of CD73 in monocytes.

This finding agrees with those reported by others confirming that collaboration with monocytes has been found to be essential for MSCs activity, from immunomodulation and Treg generation (17, 65) to stopping infiltration and aiding in the regeneration of inflamed tissue (19, 23, 24, 66).

Noteworthy, since the *in vitro* co-culture setting and the *in vivo* local administration of MSCs hinders the distinction between monocyte- and MSC-derived soluble CD73, we did not check for presence of soluble CD73, which is a limitation of our study. This might be underestimating the upregulation in CD73 protein expression by monocytes and thus could explain in part the differences between the mRNA and protein fold increase found in monocytes.

Our functional experiments were focused to check the functionality of CD73 upregulation on MSC-conditioned monocytes. Therefore, we studied the last enzymatic step of extracellular Ado production mediated by the ectonucleotidase CD73. Pi and Ado levels did not increase in the absence of 5′AMP, and their production was only affected by APCP while not by POM1. Thus, Pi and Ado production could be attributed to CD73-mediated 5′AMP hydrolysis and discarded the action of other ectoenzymes such as CD39 or Pi release due to FACS-related sheer stress, as all cells underwent the same FACS separation.

We exclusively analyzed the presence of the ectoenzymes mediating the canonical pathway of Ado production, given that ATP would be incremented and readily available in sites of injury and inflammation such as a post-MI tissue. Nevertheless, other enzymatic pathways leading to the extracellular availability of 5′AMP, such as the conversion of NAD^+^ by CD38/CD203a (67) may be also playing a part in MSC-conditioned monocytes.

Finally, studies on the modulation by MSC of further differentiated cells such as DCs have been proven controversial. Most studies describe MSCs able to modulate DC biology specially when added early in the differentiation process. However, in line with our observations, some authors report no effect of MSCs on later steps of differentiation or maturation process (20, 21). This seems to indicate that the MSC modulation would preferentially act on non-activated cells.

In summary, the upregulation of CD73 in MSC-conditioned monocytes emerges as an additional potential mechanism supporting the long-lasting immunomodulatory and healing effects of MSCs delivery. A better understanding of the signaling pathways triggered in monocytes by MSCs will help to better define new therapies for tissue injury and regenerative medicine.

## Ethics Statement

The study protocols were approved by the Clinical Research Ethics Committee of our institution (Comitè Ètic d’Investigació Clínica, HuGTiP, Refs. CEIC: EO-10-13, EO-10-016 and EO-12-022), and conformed to the principles outlined in the Declaration of Helsinki. Written informed consent was obtained from donors.

## Author Contributions

MM-T, SR, and FB designed the study; MM-T, SR and CG-M performed the experiments; MM-T, SR, CG-M, MF, and FB analyzed and interpreted the data; MF and AB-G gave conceptual advice; MM-T, SR, and FB wrote the manuscript; AB-G and FB: final approval of the manuscript. All authors reviewed the manuscript.

## Conflict of Interest Statement

The authors declare that the research was conducted in the absence of any commercial or financial relationships that could be construed as a potential conflict of interest.

## Abbreviations

Ab, antibody; Ado, adenosine; 5′AMP, adenosine 5′-monophosphate; ATP, adenosine triphosphate; cATMSCs, adipose tissue-derived MSCs; CD39, nucleoside triphosphate diphosphohydrolase; CD73, ecto-5′-nucleotidase; DAMPs, damage-associated molecular patterns; DAPI, 4′,6-diamidino-2-phenylindole dihydrochloride; DC, dendritic cell; HGF, hepatic growth factor; HLA-G, human leukocyte antigen-G; HO-1, heme oxygenase-1; IDO, indoleamine 2,3-dioxygenase; IL6, interleukin 6; MDDC, monocyte-derived dendritic cell; MI, myocardial infarction; MSC, mesenchymal stem cell; NO, nitric oxide; PGE2, prostaglandin E2; TGFβ, transforming growth factor-β; TNFα, tumor necrosis factor α; UCMSCs, umbilical cord MSCs.
