# Supplementary material for: Mesenchymal Stem Cells Induce Expression of CD73 in Human Monocytes In Vitro and in a Swine Model of Myocardial Infarction In Vivo
Source: Front Immunol. 2017 Nov 20;8:1577. doi: 10.3389/fimmu.2017.01577 (PMC5701925; doi:10.3389/fimmu.2017.01577)
Supplement: Supplementary file 1 [file Image_1.PDF]

Supplementary Material

Mesenchymal Stem Cells Induce Expression of CD73 in Human Monocytes *In Vitro* and in a Swine Model of Myocardial Infarction *In Vivo*

Marta Monguió-Tortajada, Santiago Roura, Carolina Gálvez-Montón, Marcella Franquesa, Antoni Bayes-Genis*, Francesc E. Borràs*

***Correspondence:**

Antoni Bayes-Genis: abayes@germanstrias.gencat.cat

Francesc E. Borràs: feborras@igtp.cat

Supplementary Figures and Tables:

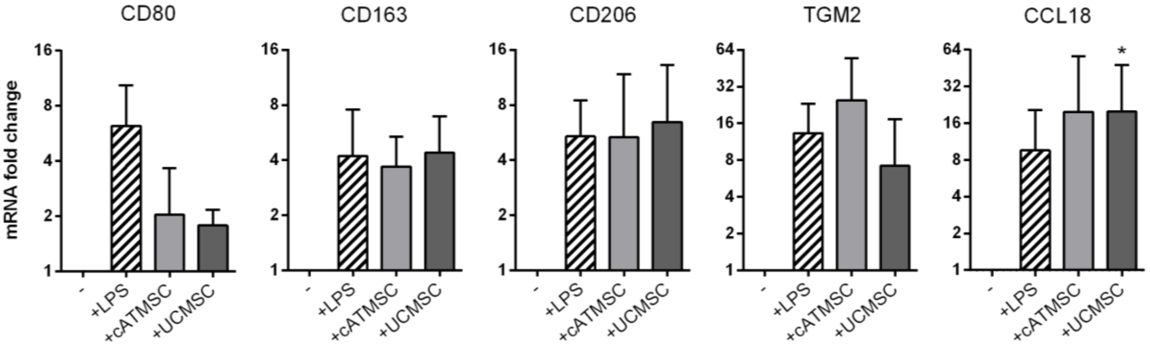

SUPPLEMENTARY FIGURE 1 | Polarization markers. mRNA fold change of the M1 marker CD80 and M2 markers CD163, CD206, TGM2 and CCL18 in monocytes stimulated with LPS or co-cultured with cATMSCs or UCMSCs compared to monocytes alone (-). Statistical differences are indicated where *$p<0.05$ to monocytes alone by One-way ANOVA with Tukey's post-hoc test.

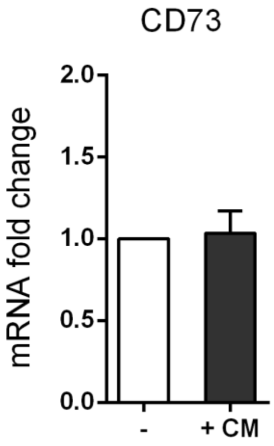

SUPPLEMENTARY FIGURE 2 | CD73 mRNA expression in monocytes is unchanged by MSC's conditioned media. mRNA fold change of CD73 (2^-ΔΔCt) of monocytes cultured in the presence of UCMSC's conditioned media (CM), relative to monocytes cultured alone. Data is expressed as mean + SD and accounts for four independent experiments of different monocyte and UCMSC donors.

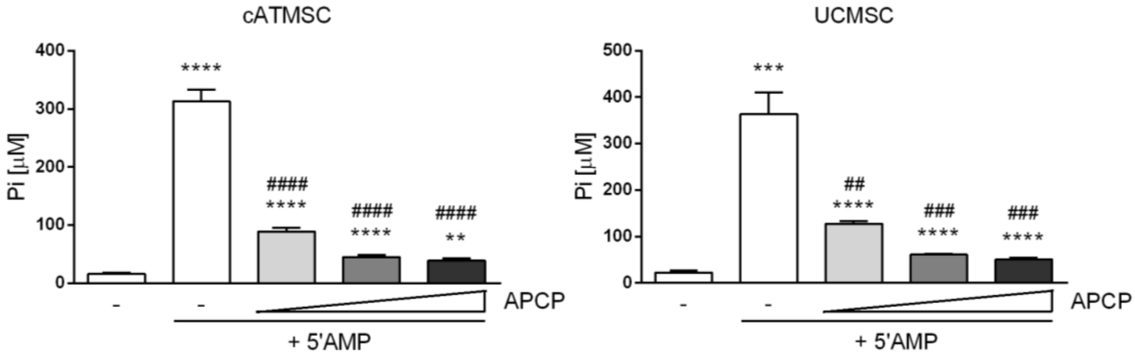

SUPPLEMENTARY FIGURE 3 | CD73 activity of MSCs. Levels of inorganic phosphate produced by cATMSCs and UCMSCs after 2h of the addition of the CD73 substrate 5'AMP (1 mM), with or without the CD73 inhibitor (APCP; 10, 50 or 100 µM). Data is represented as the mean + SD of six independent experiments. Statistical differences are indicated where **p<0.01, ***p<0.001, ****p<0.0001 compared to cells without 5'AMP by One-way ANOVA with Tukey's post-hoc test; and ##p<0.01, ###p<0.001, ####p<0.0001 compared to cells with 1mM 5'AMP and without APCP, by Student's T-test.

SUPPLEMENTARY TABLE 1 | Primers used for real time PCR:

Gene	Primer	Sequence (5'-3')	Tm [ºC]	%GC	Amplicon size [bp]
18S	Forward	TCTTTCTCGATTCCGTGGGT	58.74	50	145
	Reverse	TCTAAGAAGTTGGGGGACGC	59.39	55	
CCL18	Forward	GCTGCCTCGTCTATACCTCC	59.4	60	113
	Reverse	CCGGCCTCTCTTGGTTAGGA	60.98	60	
CD80	Forward	CTGCCTGACCTACTGCTTTG	58	55	77
	Reverse	GGCGTACACTTTCCCTTCTC	58	55	
CD163	Forward	CACCAGTTCTCTTGGAGGAACA	59	50	82
	Reverse	TTTCACTTCCACTCTCCCGC	59	55	
CD206	Forward	ACACAAACTGGGGGAAAGGTT	59.99	47.62	174
	Reverse	TCAAGGAAGGGTCGGATCG	58.8	57.89	
IL10	Forward	CGAGATGCCTTCAGCAGAGT	59.82	55	189
	Reverse	CGCCTTGATGTCTGGGTCTT	60.04	55	
TGM2	Forward	CCTCGTGGAGCCAGTTATCAA	59	52	223
	Reverse	GTCTGGGATCTCCACCGTCTTC	62	59	
